# Preoperative Neutrophil‐to‐Lymphocyte Ratio Predicts Recurrence in HPV‐Associated Oropharyngeal Cancer After Transoral Robotic Surgery

**DOI:** 10.1002/ohn.70328

**Published:** 2026-06-29

**Authors:** Micah K. Harris, Kelly Daniels, Vanessa Helou, Katie Carlson, Shivam D. Patel, Elizabeth Sell, Matthew E. Spector, Jose P. Zevallos, Joshua D. Smith, Jessica H. Maxwell, Seungwon Kim, Shaum S. Sridharan, Kevin J. Contrera

**Affiliations:** ^1^ Department of Otolaryngology–Head and Neck Surgery University of Pittsburgh Medical Center Pittsburgh Pennsylvania USA

**Keywords:** biomarkers, HPV, NLR, oropharyngeal SCC, TORS

## Abstract

**Objective:**

Evaluate the role of preoperative neutrophil‐to‐lymphocyte ratio (NLR) as a predictor of survival outcomes after transoral robotic surgery (TORS) for human papillomavirus‐related (HPV+) oropharyngeal squamous cell carcinoma (OPSCC).

**Study Design:**

Single‐institution retrospective review of patients who underwent TORS for HPV + OPSCC from April 2014 to October 2021 and had a preoperative complete blood count test.

**Setting:**

Tertiary academic center.

**Methods:**

Preoperative NLR was calculated from complete blood counts drawn within 1 month prior to surgery. Receiver operating characteristic curves evaluated performance of prognostic markers. Kaplan‐Meier estimator and Cox regression analyses were used to evaluate survival outcomes when controlling for confounding variables.

**Results:**

A total of 117 patients were included (85.5% male, 94.0% Caucasian, 59.6 ± 9.1 years old), of which 13 patients (11.1%) recurred at a median of 23.5 ± 23 months, including 7 distant metastases. At the latest follow‐up, 90.6% remained alive. On Kaplan‐Meier analyses, a preoperative NLR of >2.2 was associated with worse recurrence‐free survival (77.1% vs 95.5%, *P* = .02), whereas overall survival was similar between patients (*P* = .94). When controlling for demographics, tumor stage, pathologic risk criteria, and adjuvant therapy, NLR > 2.2 remained a significant independent predictor of recurrence on multivariate Cox regression analysis (HR 20.6, 95% CI 1.92‐80.7, *P* = .012).

**Conclusion:**

While OS was similar across groups, a preoperative NLR of >2.2 predicted worse recurrence‐free survival in a cohort of patients with HPV + OPSCC who underwent TORS. Future studies may consider integrating NLR into prospective trials as a complementary risk‐stratification biomarker.

Human papillomavirus‐related (HPV+) oropharyngeal squamous cell carcinoma (OPSCC) is a distinct disease entity that tends to carry a favorable prognosis. Early‐stage tumors are often managed with upfront resection by transoral robotic surgery (TORS) followed by radiotherapy (RT) or chemoradiotherapy (CRT). Given the acute and long‐term toxicity of RT, de‐escalation has been a major focus of recent and ongoing clinical trials.^1^, ^2^ Criteria for reduced RT include early‐stage tumors, favorable pathologic features, low smoking history, or response to induction chemotherapy.^2^ However, many of these criteria can be subjective and imprecise, highlighting a space for additional biomarkers.

Neutrophil‐to‐lymphocyte ratio (NLR) has emerged as a novel biomarker of cancer‐related inflammation and survival outcomes for many different cancers, including head and neck tumors.^3^ This is based on the underlying hypothesis that systemic inflammation reflects changes in the tumor inflammatory microenvironment, with a high NLR reflecting overactivation of the innate rater than the adapative immune system.^3^ Similar markers based on complete blood count (CBC) have been proposed, including platelet‐to‐lymphocyte ratio (PLR), lymphocyte‐to‐monocyte ratio (LMR), and pan‐immune‐inflammation value (PIV), although these have yielded mixed results.^4^

Pretreatment NLR has been shown to prognosticate survival outcomes in patients treated with definitive RT or CRT for HPV + OPSCC. However, patients treated with surgery make up a minority of subjects within these cohorts.^5^, ^6^ To date, the prognostic implications of NLR and other CBC‐derived inflammatory markers have not been investigated in a cohort of TORS patients. A better understanding of these biomarkers is highly relevant in this surgical population, as it may help providers better determine adjuvant therapy and may be integrated into clinical trials focused on treatment de‐escalation.

## Methods

A retrospective chart review of patients who underwent TORS for HPV + OPSCC at the University of Pittsburgh Medical Center from April 2014 to October 2021 was performed. Approval was obtained through the University of Pittsburgh Medical Center institutional review board (IRB# CR20030085‐029). All patients were included if they were 18 years or older, had pathologically‐confirmed HPV+ or p16+ primary OPSCC, underwent TORS for HPV + OPSCC, and had peripheral blood CBC drawn within 30 days prior to surgery. Patients were excluded if they had received steroids within 2 weeks of peripheral blood CBC, had immunocompromised conditions, were undergoing immunomodulatory treatment, underwent neoadjuvant immunotherapy, or had hematogenous pathologies that would alter inflammation. Tumor HPV status was determined using p16 immunostaining or HPV DNA detection methods. Treatment was determined based on tumor stage, pathologic risk factors, and clinician judgment.

Demographic and clinicopathologic characteristics were obtained from chart review, including sex, age, race, smoking history, alcohol history, adjuvant RT or CRT, primary tumor site, pathologic T and N classification (AJCC 8th edition), and pathologic risk features. Advanced T category was defined as pathologic T2‐3 and compared against pathologic T1 tumors. Advanced N category was defined as N2 disease. Overall survival (OS) was defined as the time from surgery to the date of death or censor. Recurrence‐free survival (RFS) was defined as the time from surgery to the time of the first detection of any recurrence (clinical exam or imaging) or censor. Multivariate Cox Regression analyses to predict recurrence and death were designed including gender, age, alcohol and smoking history, adjuvant RT, advanced T (T2‐3) and N (N2) categories, positive margins, extranodal extension, perineural invasion, angiolymphatic invasion, and NLR.

Preoperative NLR was obtained by dividing the total pretreatment neutrophil count by the total pretreatment lymphocyte count per patient, PLR by dividing the total pretreatment platelet count by the total pretreatment lymphocyte count, LMR by dividing the total pretreatment lymphocyte count by the total pretreatment monocyte count, and PIV by multiplying total pretreatment neutrophil, platelet, and monocyte counts, then dividing by total pretreatment lymphocyte count. Optimal cut‐offs were determined using receiver operating characteristic (ROC) analyses and Youden indices.

### Statistical Analysis

ROC curves were designed to determine sensitivity, specificity, and area under the ROC curve (AUC). Youden indices were used to select the optimum cut‐offs. Categorical variables were compared using chi‐square tests, and continuous variables were compared using unpaired *t*‐tests. One‐way analysis of variance compared >2 categorical variables. Cox regression models were used for proportional risk assessment in univariate and multivariate analyses, and hazard ratios (HRs) and 95% confidence intervals (CIs) were used to report the magnitude of the differences and the strength of association. Kaplan‐Meier curve analysis was used to evaluate NLR as a predictor of OS and RFS. Statistical analyses were performed with SPSS Statistics version 28.0 (IBM). Figures were created using R version 4.5.2 (R Foundation for Statistical Computing). Statistical significance was defined as a *P* < .05.

## Results

### Demographics and Clinical Data

Demographics and clinical data are outlined in Table 1. A total of 117 patients (85.5% male, 94% Caucasian, 59.6 ± 9.1 years old) who underwent TORS for HPV + OPSCC from April 2014 to October 2021 were included. All patients had peripheral blood CBC collected within 30 days prior to surgery. Average NLR for the cohort was 3.09 ± 3.5 (range: 0.16‐29.6).

**Table 1:** Demographics and Clinical Variables

Variable	Total (n = 117)	Low NLR (≤2.2) (n = 61)	High NLR (>2.2) (n = 73)	*P* value
Age (y)	59.6 ± 9.1	59.7 ± 9.5	59.5 ± 8.9	.89
Male (n, %)	100 (85.5)	45 (83.3)	55 (87.3)	.73
Caucasian, n (%)	110 (94)	49 (90.7)	61 (96.8)	.32
Smoking history, n (%)	78 (66.7)	36 (66.7)	42 (66.7)	>.99
Alcohol use history, n (%)	18 (15.4)	9 (16.7)	9 (14.3)	.92
Concurrent neck dissection, n (%)	117 (100)	61 (100)	73 (100)	n/a
HPV or p16 positive, n (%)	117 (100)	61 (100)	73 (100)	n/a
Advanced T category, n (%)	58 (49.6)	31 (57.4)	27 (42.9)	.17
Advanced N category, n (%)	44 (37.6)	23 (42.6)	21 (33.3)	.4
Positive margins, n (%)	30 (25.6)	12 (22.2)	18 (28.6)	.57
Perineural invasion, n (%)	12 (10.3)	4 (7.4)	8 (12.7)	.53
Angiolymphatic invasion, n (%)	47 (40.2)	24 (44.4)	23 (36.5)	.49
Extranodal extension, n (%)	38 (32.5)	21 (38.9)	17 (27)	.24
Adjuvant RT, n (%)	75 (64.1)	37 (68.5)	38 (60.3)	.47
Adjuvant CRT, n (%)	43 (36.8)	22 (40.7)	21 (33.3)	.53
Disease‐specific follow‐up	55.7 ± 36.4	57.1 ± 33	54.6 ± 39.3	.72
Any follow‐up	74 ± 37.1	73.2 ± 34.4	74.6 ± 39.6	.84

Abbreviations: CRT, chemoradiation therapy; HPV+, human papillomavirus positive; N, nodal; NLR, neutrophil‐to‐lymphocyte ratio; RT, radiation therapy; T, tumor; y, years.

A total of 78 patients (66.7%) had a smoking history at diagnosis, and 18 patients (15.4%) had an alcohol use history. At the time of TORS, all patients (100%) underwent concurrent neck dissection. Based on the AJCC 8th edition for pathologic staging of HPV + OPSCC, 59 patients (50.4%) had T1, 52 (44.4%) had T2, and 6 (5.2%) had T3 tumors. Fourteen patients (12%) had N0, 59 (50.4%), and 44 (37.6%) had N2 tumors. Patients were separated into advanced pathologic T category (T2‐3) (58 patients, 49.6%) and advanced pathologic N category (N2) (44 patients, 37.6%). On final pathology, 30 patients (25.6%) had positive tumor margins, 12 (10.3%) had perineural invasion, 47 (40.2%) had angiolymphatic invasion, and 38 (32.5%) had at least microscopic extranodal extension. Postoperatively, 75 patients (64.1%) completed adjuvant RT and 43 (36.8%) completed CRT. The mean follow‐up with any provider was 74 ± 37.1 months. The mean duration of disease‐specific follow‐up (with an otolaryngologist, radiation oncologist, or medical oncologist) was 55.7 ± 36.4 months. All patients had at least 3 years of disease‐specific follow‐up or recurred within 3 years.

### NLR and RFS

A total of 13 patients (11.1%) recurred at a mean of 23.5 ± 23 months, including 7 distant metastases. Overall, 10 patients recurred within 3 years after surgery and 12 patients within 5 years. The average NLR of those who recurred was 3.4 ± 2 versus 3.1 ± 3.7 for those who did not (*P* = .59). Recurrence‐free survival was 93.2% at 1 year, 91.5% at 3 years, and 89.7% at 5 years.

Using ROC curve analysis (Youden indices), an NLR cutoff of 2.2 was identified as the optimal predictor of RFS (AUC 0.65, sensitivity 84.6%, specificity 50%). Patients were then classified into low NLR (≤2.2) versus high NLR (>2.2) groups. Overall, 63 patients (53.8%) had a high preoperative NLR. Demographics and clinicopathologic characteristics of high versus low NLR groups were statistically similar (Table 1). Of those who recurred, 84.6% had a high (>2.2) preoperative NLR versus 50% for those who did not recur (*P* = .02).

**Figure 1:**
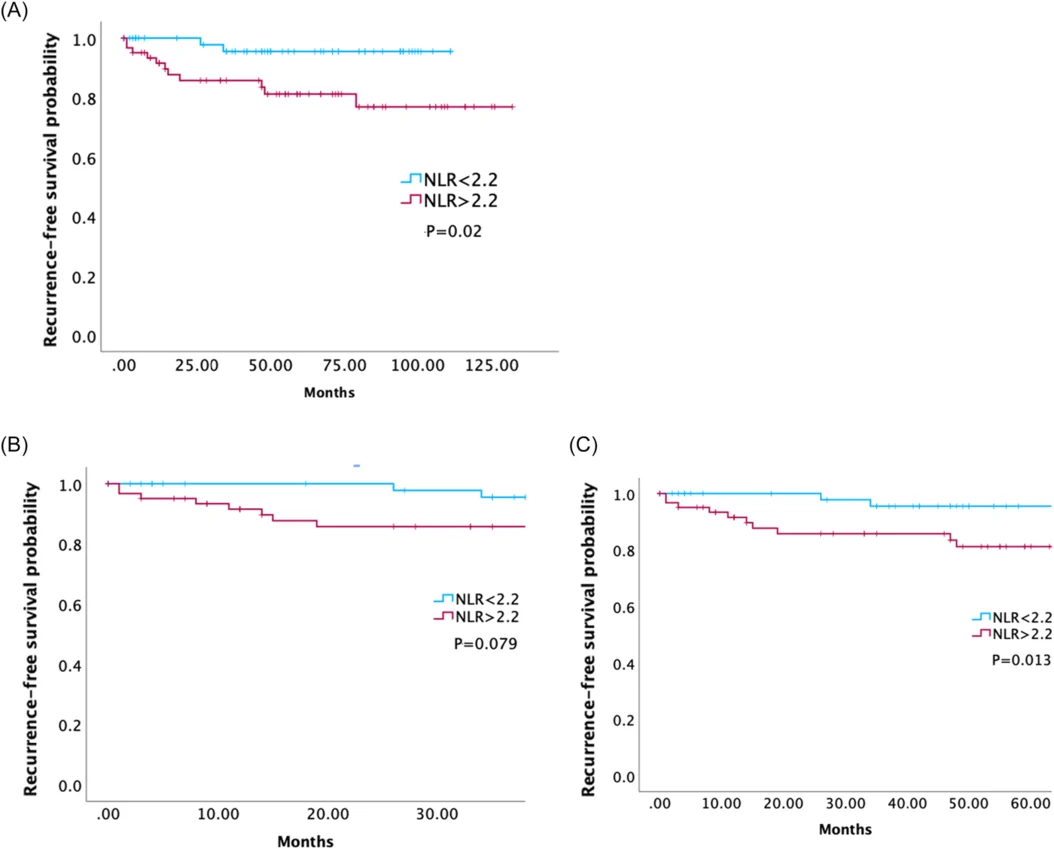
Kaplan‐Meier survival curves for (A) overall, (B) 3‐year, and (C) 5‐year recurrence‐free survival.

On Kaplan‐Meier curve analysis, those with preoperative NLR > 2.2 had worse overall RFS (77.1% vs 95.5%, *P* = .02) and 5‐year RFS (81.4% vs 95.5%, *P* = .031), with 3‐year RFS nearly reaching significance (86% vs 95.5%, *P* = .079) (Figure 1). On univariate Cox regression analysis, NLR > 2.2 was significantly associated with time to recurrence (HR 5, 95% CI 1.11‐22.59, *P* = .036). On a multivariate model, NLR > 2.2 remained an independent prognostic factor for recurrence (HR 20.6, 95% CI 1.92‐80.7, *P* = .012) while controlling for gender, age, alcohol and smoking history, adjuvant RT, advanced T (T2‐3) and N (N2) categories, positive margins, extranodal extension, perineural invasion, and angiolymphatic invasion (Table 2).

**Table 2:** Cox Regression Analysis to Predict Time to Recurrence

	Univariate analysis			Multivariate analysis		
Variable	HR	95% CI	*P*‐value	HR	95% CI	*P*‐value
Male gender	1.66	0.21‐12.96	.63	1.9	0.12‐30.15	.65
Age (y)	0.96	0.89‐1.03	.23	0.94	0.86‐1.03	.16
Alcohol history	1.15	0.25‐5.35	.86	1.88	0.23‐15.13	.55
**Smoking history**	0.3	0.09‐1.04	.057	**0.2**	**0.04‐0.9**	**.042**
Adjuvant RT	2.29	0.5‐10.6	.29	3.15	0.54‐18.6	.2
Advanced T Category	0.61	0.18‐2.07	.42	2.24	0.43‐11.61	.34
Advanced N Category	1.21	0.37‐3.97	.75	2.42	0.62‐9.44	.2
Positive Margins	2.86	0.87‐9.41	.08	4.57	0.88‐23.8	.07
Extranodal Extension	1.22	0.36‐4.16	.76	1.1	0.14‐8.4	.93
Perineural Invasion	1.97	0.42‐9.14	.38	1.27	0.12‐13.5	.84
Angiolymphatic Invasion	0.74	0.22‐2.55	.64	0.35	0.05‐2.34	.28
**NLR** > **2.2**	9.14	**1.11‐22.59**	**.036**	**20.6**	**1.92‐80.7**	**.012**

Bold values indicate statistical significance.

Abbreviations: CI, confidence interval; HR, hazard ratio; NLR, neutrophil‐to‐lymphocyte ratio; RT, radiation therapy; y, years.

### NLR and OS

At last follow‐up, 107 patients (90.6%) of patients were alive. OS was 94% and 91.5% at 3 and 5 years, respectively. The average NLR was not significantly different between patients who were alive (3.2 ± 3.7) and those who were deceased at latest follow‐up (2.4 ± 1.1, *P* = .13). On ROC curve analysis, an NLR of 2.4 was found to be most predictive of survival, though this had poor predictive strength (AUC 0.46, sensitivity 45.5%, specificity 55.7%). Moreover, OS was similar between those with an NLR of >2.4 (9.6%) versus ≤2.4 (9.2%, *P* = .94).

**Figure 2:**
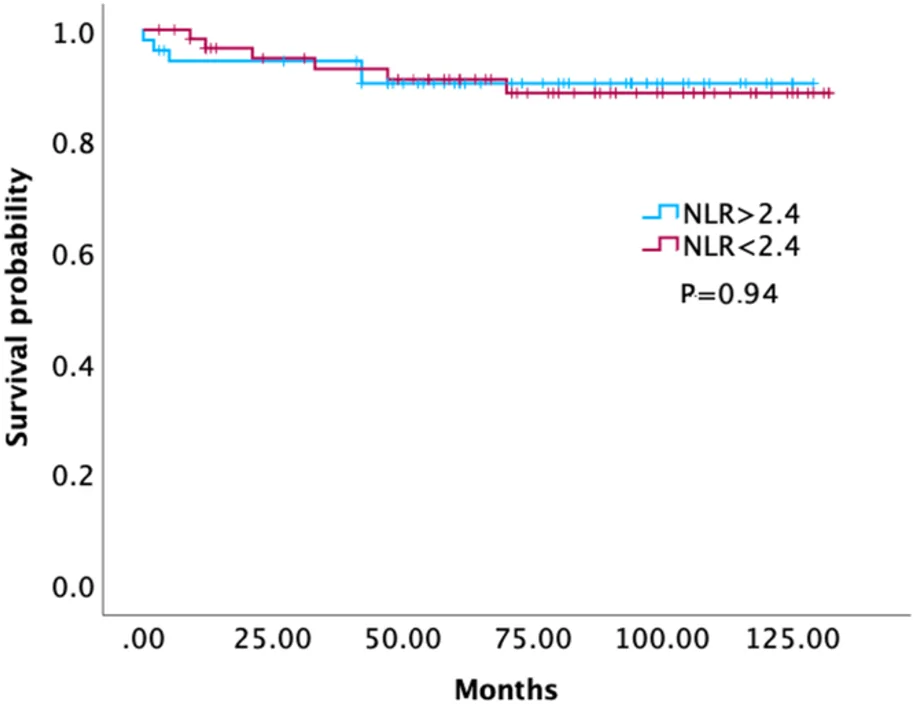
Overall survival stratified by NLR. NLR, neutrophil‐to‐lymphocyte ratio.

On Kaplan‐Meier curve analysis, survival was statistically similar between those with preoperative NLR of >2.4 versus ≤2.4 (*P* = .94) (Figure 2). On univariate and multivariate Cox regression analyses, NLR > 2.2 was not a significant predictor of time to death. Gender, age, alcohol and smoking history, adjuvant RT, advanced T (T2‐3) and N (N2) categories, positive margins, extranodal extension, perineural invasion, and angiolymphatic invasion were also insignificant on multivariate analysis (Table 3).

**Table 3:** Cox regression analysis to predict time to death

	Univariate analysis			Multivariate analysis		
Variable	HR	95% CI	*P* value	HR	95% CI	*P* value
Male gender	1.2	0.21‐9.2	.52	1.7	0.12‐10.2	.6
Age (y)	1.03	0.97‐1.11	.35	1.04	0.94‐1.16	.42
Alcohol history	0.73	0.09‐5.92	.78	0.79	0.07‐8.75	.85
Smoking history	3.8	0.47‐31.2	.21	1.9	0.12‐5.06	.79
Adjuvant RT	0.87	0.21‐3.63	.85	0.63	0.12‐3.23	.58
Advanced T category	3.1	0.63‐15.4	.17	2.01	0.32‐13.5	.44
Advanced N category	1.65	0.41‐6.6	.48	0.77	0.12‐5.06	.79
Positive margins	**5.6**	**1.34‐23.46**	**.018**	3.42	0.55‐21.2	.186
Extranodal extension	2.22	0.56‐8.9	.26	1.6	0.31‐8.22	.57
Perineural invasion	1.18	0.15‐9.6	.87	0.46	0.04‐5.16	.53
Angiolymphatic invasion	4.19	0.85‐20.78	.079	3.12	0.46‐21.06	.24
NLR > 2.2	1.47	0.35‐6.2	.597	1.38	0.28‐6.74	.69

Bold values indicate statistical significance.

Abbreviations: CI, confidence interval; HR, hazard ratio; NLR, neutrophil‐to‐lymphocyte ratio; RT, radiation therapy; y, years.

### Other Inflammatory Markers

Other CBC‐derived inflammatory markers that have been correlated with survival outcomes in head and neck cancer include PLR, LMR, and PIV. To determine if these inflammatory markers were predictive of survival outcomes, ROC curve analyses were first used to identify optimal thresholds to predict recurrence for PLR (cut‐off 11.1, AUC 0.58), LMR (cut‐off 1.8, AUC 0.42), and PIV (cut‐off 470, AUC 0.53). The same was performed to predict OS for PLR (cut‐off 10.4, AUC 0.43), LMR (cut‐off 1.6, AUC 0.51), and PIV (cut‐off 129, AUC 0.36). However, using these cut‐offs, survival and recurrence rates remained statistically similar between the groups for PLR, LMR, and PIV (all *P* > .05) Thus, these markers were excluded from further analysis.

## Discussion

While inflammatory biomarkers such as NLR have been found to prognosticate survival outcomes in patients with HPV + OPSCC undergoing definitive radiotherapy or chemoradiotherapy,^6^, ^7^ there is a paucity of data regarding their role in the setting of surgery. Here, we demonstrate that a preoperative NLR of >2.2 independently predicted recurrence in a cohort of 117 patients who underwent TORS for HPV + OPSCC. This provides evidence that a cost‐effective marker such as NLR may have the potential to help guide adjuvant therapy following TORS.

The inflammatory microenvironment is a critical component in most tumors, playing a key role in driving tumor development, metastasis, and resistance.^8^, ^9^ An abundance of evidence has emerged showing that markers of systemic inflammation, such as elevated NLR, predict disease outcomes across many cancers. This is hypothesized to be due to overactivation of the innate rather than the adaptative immune system, as neutrophilia has been known to inhibit the cytotoxic activity of lymphocytes.^10^, ^11^, ^12^ Within OPSCC, pre‐treatment NLR has been shown to prognosticate survival outcomes in patients undergoing primary radiotherapy and chemoradiotherapy.^6^, ^7^ Our study builds on existing literature to apply this tool to surgical patients, demonstrating that NLR prior to surgery prognosticates recurrence. While disease stage, pathologic risk criteria, and the use of adjuvant therapy were expectedly heterogenous in our cohort, NLR > 2.2 remained an independent predictor of recurrence (HR 20.6) when controlling for these confounders on multivariate regression. Although we did not find a difference in OS, the relationship between NLR and OS is variable within the literature.^12^

These results support the growing notion that NLR may have the potential to be incorporated into clinical decision‐making. Over recent years, there has been a major focus on treatment deintensification for HPV + OPSCC. Following TORS and neck dissection, adjuvant RT or CRT is traditionally determined by pathologic risk factors. Unlike pathologic risk factors, which can be subjective and vary between pathologists, markers such as NLR are automated, objective values. Moreover, NLR holds the advantage of being a cost‐effective and accessible biomarker that requires no additional specialized testing, unlike genomic or molecular markers. As personalized treatment become more commonplace, accessible markers may become relevant for centers with less access to specialized molecular testing. Moreover, the preoperative setting is unique, as markers derived during this time are helpful to counsel patients and to discuss adjuvant treatment plans ahead of time. For example, a low preoperative NLR could be integrated into tumor board discussions to advocate for reductions in adjuvant therapy intensity (ie, 50 Gy instead of 60 Gy), or closer monitoring in those with high NLR. The accessibility of NLR may also open the field to examine these markers in other treatment paradigms where frequent monitoring of treatment response is critical, such as in the setting of neoadjuvant immunotherapy.

Future research is needed to determine appropriate dichotomized cut‐off values for specific patient groups. A multitude of studies have provided convincing evidence for NLR as an independent prognostic biomarker. However, translation into clinical practice has been hindered in part by the heterogeneity of prior study cohorts and a wide range of reported cut‐off values.^13^ In the present study, we identified an NLR cut‐off of 2.2 based on ROC curve analysis within a specific population of HPV + OPSCC that underwent TORS. In a systematic review and meta‐analysis of 10,072 head and neck cancer patients across 33 cohorts, Tham et al identified NLR cutoffs ranging from 1.9 to 5.0 for disease‐specific survival and 1.9 to 5.6 for OS.^14^ In a study of 848 OPSCC patients treated with definitive RT, Ng et al showed that a preradiation NLR of 3.0 prognosticated 5‐year OS and RFS; So et al examined a more heterogenous cohort including both surgically‐treated and radiation‐treated patients, identifying a pretreatment NLR cutoff of 2.42.^6^, ^7^ While multi‐institutional or national database studies offer larger sample sizes, the absence of standardized treatments or the exclusion of pretreatment inflammatory conditions may limit the clinical application to specific populations. Many variables can change NLR, including induction chemotherapy,^15^, ^16^ immunotherapy, HPV,^17^ or demographical factors such as racial background, gender, and age.^18^ In order to identify NLR cut‐offs that can be applied to clinical practice, future studies should seek to amass large groups of patients with very specific disease profiles and similar treatment regimens.

Broadly, these data show that while NLR and other inflammatory markers have the potential to be clinically applicable prognostic markers in head and neck cancer, further research is needed before incorporation into clinical trials. Our study is not without limitations. As a single‐institution retrospective cohort study, our findings may not be generalizable to the overall population of HPV + OPSCC patients. Second, our timeframe for preoperative CBCs was within 30 days prior to surgery, rather than a standardized timepoint. Although patients who received preoperative steroids or neoadjuvant immunotherapy were excluded, other variables that could affect neutrophil counts (such as subclinical infection) were not documented at the time of blood draw. Finally, our study cohort was relatively small, which may underpower our ability to identify cut‐off values and limit external applicability. We therefore view these results as hypothesis‐generating, providing a foundation for future multi‐institutional prospective trials to validate the NLR as a robust risk‐stratification tool in the surgical setting.

## Conclusions

In this cohort of patients with HPV + OPSCC who underwent TORS, we found that a preoperative NLR of >2.2 was a significant independent prognosticator of recurrence. While OS was similar across groups, the robust association with recurrence highlights the potential of NLR to be explored as a cost‐effective biomarker of inflammation. Future studies may consider integrating NLR into clinical trials as a complementary biomarker that can be used to further refine risk‐stratification in intermediate‐risk populations.

## Author Contributions

**Micah K. Harris**, concept and design, data acquisition and analysis, drafting and review of manuscript, final approval of version to be published, and agreement to be accountable for all aspects of the work; **Kelly Daniels**, concept and design, drafting and review of manuscript, final approval of version to be published, and agreement to be accountable for all aspects of the work; **Vanessa Helou**, concept and design, drafting and review of manuscript, final approval of version to be published, and agreement agreement to be accountable for all aspects of the work; **Katie Carlson**, data acquisition and analysis, drafting and review of manuscript, final approval of version to be published, and agreement to be accountable for all aspects of the work; **Shivam D. Patel**, data acquisition and analysis, drafting and review of manuscript, final approval of version to be published, and agreement to be accountable for all aspects of the work; **Elizabeth Sell**, data acquisition and analysis, drafting and review of manuscript, final approval of version to be published, and agreement to be accountable for all aspects of the work; **Matthew E. Spector**, data acquisition and analysis, drafting and review of manuscript, final approval of version to be published, and agreement to be accountable for all aspects of the work; **Shaum S. Sridharan**, data acquisition and analysis, drafting and review of manuscript, final approval of version to be published, and agreement to be accountable for all aspects of the work; **Jose P. Zevallos**, concept and design, drafting and review of manuscript, final approval of version to be published, and agreement to be accountable for all aspects of the work; **Joshua D. Smith**, concept and design, data acquisition and analysis, drafting and review of manuscript, final approval of version to be published, and agreement to be accountable for all aspects of the work; **Jessica H. Maxwell**, concept and design, drafting and review of manuscript, final approval of version to be published, and agreement to be accountable for all aspects of the work; **Seungwon Kim**, concept and design, drafting and review of manuscript, final approval of version to be published, and agreement to be accountable for all aspects of the work; **Kevin J. Contrera**, concept and design, drafting and review of manuscript, final approval of version to be published, and agreement to be accountable for all aspects of the work.

## Disclosures

### Competing interests

None.

### Funding source

This study was funded by National Institutes of Health (1KL2TR001856), American College of Surgeons Martin Faculty Research Fellowship, and National Cancer Institute (P50CA097190 for CA230319). Dr. Contrera reports research grant support from Droplet Biosciences, Haystack Oncology, and Natera.
